# Surfactant protein D alleviates chondrocytes senescence by upregulating SIRT3/SOD2 pathway in osteoarthritis

**DOI:** 10.1186/s10020-025-01221-6

**Published:** 2025-04-30

**Authors:** Huanyu Jiang, Yantao Zhang, Piyao Ji, Jianghua Ming, Yaming Li, Yan Zhou

**Affiliations:** 1 Department of Orthopedics, Renmin Hospital of Wuhan University, Wuhan, 430060 China; 2 Central Laboratory, Renmin Hospital of Wuhan University, Wuhan, 430060 China

**Keywords:** Surfactant protein D, Osteoarthritis, Chondrocyte, Senescence, Superoxide dismutase, Sirtuin, Mitochondria

## Abstract

**Background:**

Osteoarthritis (OA) is an age-related degenerative disease that affects bones and joints. The hallmark pathogenesis of OA is associated with chondrocyte senescence. Surfactant protein D (SP-D) is a member of the innate immune proteins family, which can inhibit the immune inflammatory response of chondrocytes. However, the effect of SP-D on chondrocyte senescence phenotype is poorly studied. The present study investigated the phenotypic regulation of OA chondrocyte senescence mediated by SP-D and explored the underlying molecular mechanism.

**Methods:**

In this study, an in vitro senescence chondrocyte model was generated by subjecting chondrocytes to IL-1β treatment. Furthermore, the expression of aging-related biomarkers and mitochondrial functions in SP-D overexpressing chondrocytes was observed. Co-immunoprecipitation was conducted to verify the association between SP-D and the identifed proteins within chondrocytes. Moreover, a rat OA model was established by destabilization of the medial meniscus surgery, and the effect of SP-D on reversing the aging phenotype of OA cartilage was investigated.

**Results:**

The results indicated that SP-D significantly decreased senescence and enhanced mitochondrial functions in senescent chondrocytes. The RNA-sequencing analysis revealed that the SIRT3/SOD2 pathway predominantly modulated the effect of SP-D on alleviating senescence. In addition, SP-D overexpression mitigated chondrocyte senescence, suppressed senescence-associated secretory phenotype (SASP) secretion and ameliorated mitochondrial damage. In the rat OA model, SP-D inhibited aging-related pathological changes by upregulating SIRT3/SOD2 pathway, thereby protecting the cartilage tissue integrity.

**Conclusion:**

These findings indicate that SP-D modulates the inhibition of chondrocyte senescence by upregulating SIRT3/SOD2 pathway. These data indicate that targeting SP-D and the SIRT3/SOD2 pathway might be a promising therapeutic strategy for OA.

**Supplementary Information:**

The online version contains supplementary material available at 10.1186/s10020-025-01221-6.

## Graphical Abstract

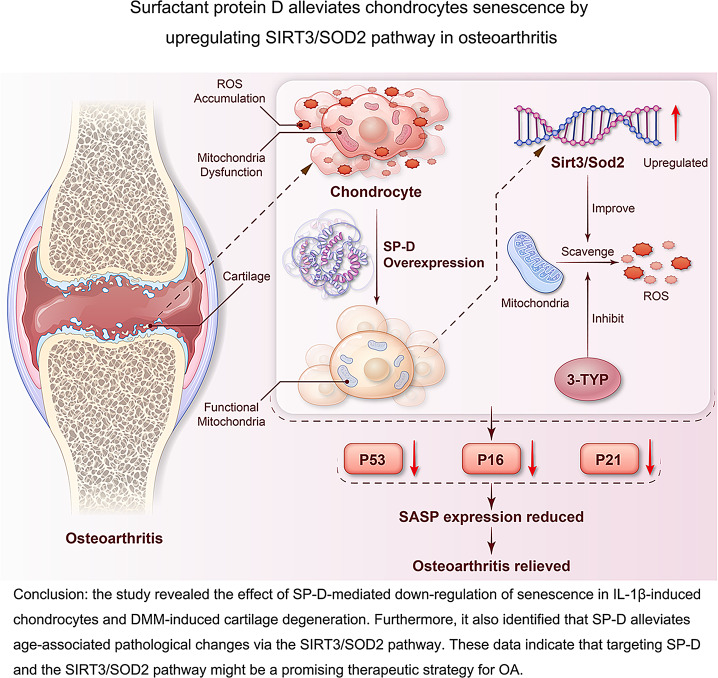

## Background

Osteoarthritis (OA) is one of the most prevalent bone and joint degenerative diseases (Bortoluzzi et al. 2018). It is characterized by the deterioration of the joint’s structure, accompanied by pathological alterations such as synovial inflammation (Mathiessen and Conaghan 2017), cartilage erosion (Sherwood 2019), osteophyte formation (Nieminen et al. 2019) and subchondral bone thickening (Kijowski et al. 2020). With the aging population, the incidence of OA is also rising, which significantly impacts patients’ quality of life and imposes substantial economic burdens. Therefore, OA is a critical health concern in society that warrants significant attention (Jiang 2022).

The etiology of OA is complex, and its onset and progression are associated with various risk factors, such as increased mechanical loading, systemic inflammatory responses, joint injuries, and genetic predispositions (Ho et al. 2022; Li et al. 2013; Palazzo et al. 2016). Furthermore, cellular senescence has been indicated as a significant pathological feature in OA pathogenesis (Liu et al. 2022). Moreover, much epidemiological evidence has indicated a strong positive correlation between OA incidence and patient age (Johnson and Hunter 2014; Plotz et al. 2021; Vina and Kwoh 2018). Coryell et al. identified a correlation between OA development and an increase in the presence of senescent cells in joint tissues (Coryell et al. 2021). Moreover, McCulloch et al. indicated that the inflammatory and catabolic mediators involved in OA pathogenesis shared marked similarities with the secretory profile of the senescent cells (McCulloch et al. 2017). Overall, these data indicated that OA therapy targeting cellular senescence might be an effective strategy against early OA progression.

Cellular senescence represents an irreversible state of cell cycle arrest marked by increased activity of senescence-associated β-galactosidase (SA-β-gal) (Debacq-Chainiaux et al. 2009) and elevated expression of the senescence-associated secretory phenotype (SASP) (Freund et al. 2010). This causes excessive secretion of pro-inflammatory substances and matrix-degrading enzymes, resulting in chondrocyte damage and the loss of extracellular matrix (ECM) (Price et al. 2002), which alters the cellular microenvironment. Previous in vivo and in vitro investigations have revealed that age-related mitochondrial dysfunction and oxidative stress are significant factors associated with cellular senescence (Finkel and Holbrook 2000; Hernandez-Segura et al. 2018; Zhou et al. 2021).

The innate immune protein, SP-D, is a C-type lectin that is highly expressed in respiratory epithelial cells and is essential for the bodyʼs defense against various diseases (Madan & Kishore 2020). Our previous research revealed significantly lower SP-D expression in articular chondrocytes of OA patients compared to normal individuals. SP-D inhibits OA articular cartilage degeneration and chondrocyte’s immune-inflammatory response via its anti-inflammatory and anti-apoptotic activities (Jiang et al. 2022). However, robust evidence supporting the role and mechanism of SP-D in chondrocyte senescence is lacking, which inhibits its application as a therapeutic target for OA.

Superoxide dismutase 2 (SOD2) is an antioxidant enzyme in mitochondria, which facilitates the conversion of superoxide anion (O^2−^) radicals into oxygen and hydrogen peroxide (H_2_O_2_) through disproportionation. This enzymatic action regulates O^2−^ levels and mitigates reactive oxygen species (ROS) induced damage (Miao and St Clair 2009). Sirtuin 3 (SIRT3), an upstream regulator of SOD2, is the primary mitochondrial acetyl lysine deacetylase, which directly interacts with and deacetylates SOD2. This interaction promotes SOD2 activity, which significantly influences the ROS balance (Yapryntseva et al. 2022). As individuals age, mitochondrial dysfunction increases, while SOD2 expression and activity decrease, which elevates basal O^2−^ production, resulting in increased oxidative stress and intracellular redox imbalance, ultimately fostering cellular senescence in joint tissues. Furthermore, increased intracellular ROS levels activate the nuclear factor kappa-B (NF-κB) pathway, which further increases the synthesis of inflammatory cytokines and matrix-degrading enzymes. These events culminate in the destruction of the cartilage matrix and inflammation of the synovial cartilage (Ansari et al. 2020; Sinha et al. 2013; Son et al. 2013). Therefore, interventions that can modulate SOD2 expression and activity, regulate mitochondrial function, and inhibit excess ROS might alleviate the senescent state of chondrocytes and delay OA progression (Bolduc et al. 2019; Riegger et al. 2023).

This study aimed to investigate SP-D’s ameliorative effect on senescent chondrocytes in OA and elucidate the potential molecular mechanisms. Furthermore, to evaluate how SP-D inhibits OA articular cartilage degeneration, an in vivo rat OA model was established by destabilization of the medial meniscus (DMM) surgery. Moreover, the impact of SP-D on the OA chondrocyte senescence via the SIRT3/SOD2-mediated signaling was assessed. The overall results indicated that SP-D could serve as a possible target for OA therapy.

## Materials and methods

### Reagents and antibodies

The SIRT3 inhibitor 3-TYP (#HY-108331) was obtained from MedChemExpress (New Jersey, USA). P16 (#R23896) antibodies were obtained from Zen-Bioscience (Chengdu, China). P21 (#28248-1-AP), P53 (#10442-1-AP), Bcl-2 (#26593-1-AP), SIRT3 (#10099-1-AP), SOD2 (#24127-1-AP), and TOM20 (#11802-1-AP) antibodies were obtained from Proteintech Group (Wuhan, China). Beta-Tubulin (#T0023) antibody was obtained from Affinity Biosciences (Cincinnati, USA). SP-D (#ab220422) and anti-SOD2/MnSOD-acetyl K68 (Ac-SOD2) (#ab137037) antibodies were obtained from Abcam (Cambridge, UK). Recombinant human SP-D (rhSP-D) (#CSB-YP021175HU) was purchased from Huamei Biotech Co., Ltd (Wuhan, China, link: https://www.cusabio.cn/Recombinant-Proteins/Recombinant-Human-Pulmonary-surfactant-associated-protein-D(SFTPD)-11865.html#a01). Rat interleukin-1 beta (IL-1β) (#Z03014) was purchased from Genscript Biotech Corporation (Nanjing, China). All of the other chemicals and reagents were of analytical grade.

## Cell culture, RNA isolation and sample preparation

Primary articular chondrocytes were isolated from the knee joints of newborn (5 days old) Sprague-Dawley (SD) rats (from the Center for Animal Experiment/ABSL-III Laboratory of Wuhan University, Wuhan, China). Third passage rat chondrocytes were cultured in 6-well plates for 12 h. The SP-D geneʼs complete cDNA length was cloned into the pcDNA3.1 vector (Youbio Biotech, China) utilizing the hot fusion method designed with CE Design V1.04 (Vazyme Biotech, China). Each primer comprised gene-specific and 17–30 bp sequences of the pcDNA3.1 vector, which contained a FLAG tag (Sigma, USA). The vector was utilized as a tagged protein and was fused with the 3ʼ end of SP-D. Upon 80% confluency, the cell culture medium was discarded and the cells were co-treated with lipofectamine 2000 transfected pcDNA3.1 empty plasmid and pcDNA3.1-SP-D plasmid (1 mg/mL). Then, after 6 h, the medium was refreshed again and the cells were incubated further for 48 h before the sample collection. The total RNA of chondrocytes was extracted using the GenElute™ Mammalian Total RNA Miniprep kit (Sigma, USA) and resolved with DNAse I (Qiagen, Germany) with 2100 Microbial Detector (Agilent Technologies, USA). Then, the extracted RNA was quantified and reverse transcribed (RNA = 150 ng/test sample) into cDNA for the reverse transcription-polymerase chain reaction (RT-PCR) using TaqMan reverse transcription reagents and a hexamer (Thermo Fisher Scientific, USA).

## RNA-sequencing and bioinformatics analysis

RNA samples were used for RNA sequencing. Briefly, total RNA was extracted, the cDNA library was prepared, and RNA transcriptome sequencing (Illumina HiSeq 4000) was performed by Shenzhen BGI Tech Co., Ltd. There were three biological replicates for both the pcDNA3.1-SP-D and control chondrocytes and six RNA-seq samples (Ctrl_1st, Ctrl_2nd, Ctrl_3rd, SPD_1st, SP-D_2nd, and SP-D_3rd) were acquired. The gene expression was presented via the fragments per kilobase of transcript per million fragments mapped (FPKM). Using the Agilent 2100 Microbial Detector (Labx, Canada), RNA features were confirmed. A template with RNA Concordance Number > 6.5 was utilized for RNA sequencing. After transcriptome sequencing, high-component unidentified base (N) noise readings and low-quality environmental contaminants from the power adapter were filtered out. The reads were then aligned to the reference gene (NCBI Rnor 6.0) using Bowtie 2 (http://bowtie-bio.sourceforge.net/Bowtie2/index.html). Subsequently, DEseq2 was employed to assess differential expression genes (DEGs). The adjusted p-value was < 0.05 and the fold change was > 2. To evaluate the performance levels of DEGs, a heat map was made using MeV. Furthermore, the DEGs were utilized to search the Kyoto Encyclopedia of Genes and Genomes (KEGG) and Gene Ontology (GO) databases for identifying gene functions.

## Recombinant adeno-associated virus (rAAV) construction

The shRNAs specific for SP-D/scrambled controls were cloned into the GV478 AAV vector (Genechem, China) and then co-transfected into AAV-293 cells with the pAAV-RC and pHelper vectors. The rAAV9 particles were isolated from cell supernatants, concentrated, and purified for in vivo studies.

## Animal studies

Male SD rats (age = 8 weeks) were provided by the Wuhan Center for Animal Experiments/ABSL-III Laboratory of Wuhan University. The study design was approved by the Laboratory Animal Welfare and Ethics Committee of the Renmin Hospital of Wuhan University (Approval No: WDRM20220603C) and followed the National Research Council’s Guide for the Care and Use of Laboratory Animals. For the rAAV serotype investigation of small animals with normal bones and joints, the rats were intra-articularly injected with rAAV-encoded SP-D-specific shRNA [1 × 1010 deoxyribonuclease resistant particle (drp)/25 mL/knee joint] for 10 consecutive days (Table 1). After 4 weeks of the initial injection, the treatment was terminated, and the rats were euthanized.

**Table 1:** The different treatments received by each group

Group	PBS	rAAV-GFP	rAAV-SP-D shRNA	rhSP-D	3-TYP
Control group	-	-	-	-	-
rAAV-GFP + Control group	-	+	-	-	-
rAAV-SP-D + Control group	-	-	+	-	-
Sham operation group	+	-	-	-	-
OA-induction group	+	-	-	-	-
OA + rAAV-SP-D shRNA group	-	-	+	-	-
OA + rhSP-D group	-	-	-	+	-
OA + rhSP-D + 3-TYP group	-	-	-	+	+

To acclimate the rats to the laboratory environment, all the animals were fed under standard conditions for one week. The rats were randomly categorized into five groups (*n* = 5): sham operation, OA-induction, OA + rAAV-SP-D shRNA, OA + rhSP-D, and OA + rhSP-D + 3-TYP. rAAV-SP-D shRNA is silent RNA that silences the expression of SP-D. In the sham operation group, only the joint cavity was exposed and no further surgery was performed. OA was induced in the right knee through DMM surgery (Glasson et al. 2007). After fourth-week post-surgery, rats in the relevant groups were injected weekly with rAAV-SP-D shRNA (1 × 10^10^ drp, 25 µL), and rhSP-D (0.0033 mg/kg). After 1 day, 3-TYP (100 µM, 25 µL) was injected into the appropriate group. In contrast, rats in OA-induction and sham operation groups received an injection of 50 µL of phosphate-buffered saline (PBS) into the right knee joint cavity. The animals were euthanized by cardiac exsanguination after 10 weeks post-surgery. Their knee joints were dissected, fixed with 4% paraformaldehyde for 24 h, decalcified with 10% EDTA solution for 6 weeks, and then embedded in paraffin.

### Histology analysis

Samples were sliced into 5 μm sagittal sections and stained with Hematoxylin-Eosin (H&E). Two blind observers were used to analyze the pathophysiology according to the modified Mankin’s score (Wang et al. 2014). Additionally, the samplesʼ proteoglycan levels were ascertained by Safranin-O-Fast Green stain.

## Immunofluorescence analysis

The SP-D, SIRT3, SOD2, P16, P53, matrix metalloproteinase-3 (MMP3), and Collagen II expressions in articular cartilage were evaluated utilizing immunofluorescence. Briefly, the sample slices were treated in the dark for 1 h with a fluorescent-conjugated secondary antibody (Boster Biological Technology, China) and examined using fluorescence microscopy (AX10, Carl Zeiss, Germany). The immunofluorescent mean densities of these indicators were evaluated by Image-Pro Plus 6.0 image analysis software (Media Cybernetics Co., USA).

## Mitochondria in synovial tissues analyses

Samples of synovial tissue were obtained from the iliac tendonʼs side and fixed using 2.5% Glutaraldehyde Fixative (BioSharp, China). The samples were washed with PBS, treated with 1% osmium oxide at 4 °C, immobilized for 3 h, washed with dH_2_O, dried with gradient concentration of alcohol and toluene, and then embedded in epoxy resin. The samples were double-stained and observed with a transmission electron microscope (TEM, H-600IV, HITACHI, Japan).

### Micro-CT and Micro-MRI evaluations

Knee joint images were obtained using Micro-CT (Skyscan 1176, Bruker microCT N.V., Kontich, Belgium; 4000 × 2672 pixels, 9 μm isotropic voxel). The overall structure was assessed based on the microtomographic data from 3D morphometry. Furthermore, quantitative morphometric indices [bone volume fraction (BV/TV, %), average trabecular thickness (Tb. Th, mm), and average trabecular separation (Tb. Sp, mm)] were also assessed.

MRI images were collected using a 7.0T vertical bore Bruker Biospec 70/30 scanner (BrukerBioSpin MRI GmbH, Rheinstetten, Germany). The scan settings were tailored for gray-white matter contrasts. Each image processing time was 30 min.

### Cell stimulation

The neonatal rat’s chondrocytes were cultured at 37 °C and 5% CO2 until 80% confluency. After the third generation, cells were seeded for 12 h in 6-well culture plates. The media was then aspirated, and the cells were pre-treated with pcDNA3.1-SP-D (1 mg/mL) and SIRT3 inhibitor 3-TYP (50 µM) for 2 h before a 24-hour co-treatment with IL-1β (10 ng/mL).

### SA-β-gal staining

The SA-β-Gal staining kit (Beyotime, China) was used in accordance with the manufacturerʼs instructions for SA-β-Gal staining. Senescent cell staining fixative was used to fix chondrocytes for a duration of 15 min. The chondrocytes underwent three times of PBS washing before being incubated in SA-β-Gal staining working solution for 24 h at 37 °C. The staining was examined with a fluorescence microscope (Olympus, Japan). SA-β-gal positive chondrocytes were counted in three random fields per well.

### Coimmunoprecipitation (Co-IP) assay

The regulatory effect of SP-D on SOD2 protein was analyzed by Co-IP. Chondrocytes were dissolved in a precooled buffer containing protease inhibitors, and incubated with anti-SP-D and anti-SOD2 antibodies at 4 °C overnight. They were then combined with protein A Sepharose, cultured for 1 h at 4 ℃, and rinsed with water three times. Then, western blotting was performed.

### Mitochondrial membrane potential assay

Mitochondrial membrane potential was determined using the Mitochondrial Membrane Potential (ΔΨM) assay kits with JC-1 and Rhodamine 123 (Beyotime, China), per the kit’s guide. Briefly, chondrocytes were washed and cultured in a mixture of complete DMEM/F12 (1 mL) and JC-1 working solution (1 mL) for 25 min at 37 °C. Then, the staining solution was removed and, the cells were cultured in 2 mL of complete DMEM/F12 medium. The red-green fluorescence was detected under a fluorescence microscope (Olympus, Japan), and the ratio was calculated.

For Rhodamine 123 staining, the chondrocytes were washed, and then 1 mL of Rhodamine 123 working solution was added for 25 min at 37 °C. Then, the staining solution was aspirated and 2 mL of complete DMEM/F12 medium was added. The green fluorescence was observed using a fluorescence microscope (Olympus, Japan).

### MitoTracker red staining

The morphology of chondrocytes’ mitochondria was determined using MitoTracker Red staining (Beyotime, China). Chondrocytes were first washed with PBS, followed by co-incubation of 50 nM MitoTracker Red working solution with the cells for 20 min at 37˚C. The staining solution was removed and fresh cell culture medium was added. The morphology of mitochondria was visualized using a fluorescence microscopy (Olympus, Japan).

### SOD2 activity analysis

SOD2 activity was measured using the Cu/Zn-SOD and Mn-SOD Assay Kit (Beyotime, China) according to the manufacturer’s instructions. Briefly, chondrocytes were harvested and homogenized, and supernatant was pre-treated with Cu/Zn-SOD inhibitor. Subsequently, the samples were incubated with the working solution for 30 min at 37˚C. The absorbance at 450 nm was measured using a microplate reader (PerkinElmer, USA) and the activity of SOD2 was calculated.

### Measuring ROS levels

ROS levels were measured using the Reactive Oxygen Species Assay Kit (Beyotime, China). Briefly, DCFH-DA was diluted using serum-free media (1:1000) before adding cells for 20 min at 37 °C. Then, using a fluorescence microscope (Olympus, Japan), ROS levels were determined.

### Western blotting

The total chondrocyte proteins were extracted using RIPA lysis buffer (Biosharp, China) and the concentration of proteins was measured by the BCA protein assay kit (Beyotime, China) in compliance with the manufacturerʼs guidelines. After being separated on 12% SDS-PAGE, the protein samples were transferred to a PVDF membrane and blocked with 5% skimmed milk solution for 2 h. The primary antibodies against SP-D, P16, P21, P53, Bcl-2, Tom20, SIRT3, SOD2, Ac-SOD2 and beta-tubulin were then used to probe the blots at 4 °C overnight. The blots were washed in TBST thrice, incubated with HRP-conjugated secondary antibodies for 1 h, treated with ECL substrate (Beyotime, China), and visualized using ChemiDoc Touch (Bio-Rad, USA). The relative expression level of the target protein was normalized to the band intensity of beta-tubulin.

### Statistical analysis

Data are presented as mean ± standard error of the mean (SEM). Student’s t-test and one-way analysis of variance (ANOVA) with Spearman rank correlation test were used for two-group and multi-group comparisons, respectively. All statistical analyses were performed by SPSS version 15.0 (IBM, USA) and GraphPad Prism 5 software (GraphPad Software, UK). P value < 0.05 was considered statistically significant.

## Results

### SP-D and SIRT3/SOD2 expression are significantly decreased in senescent chondrocytes

**Fig. 1:**
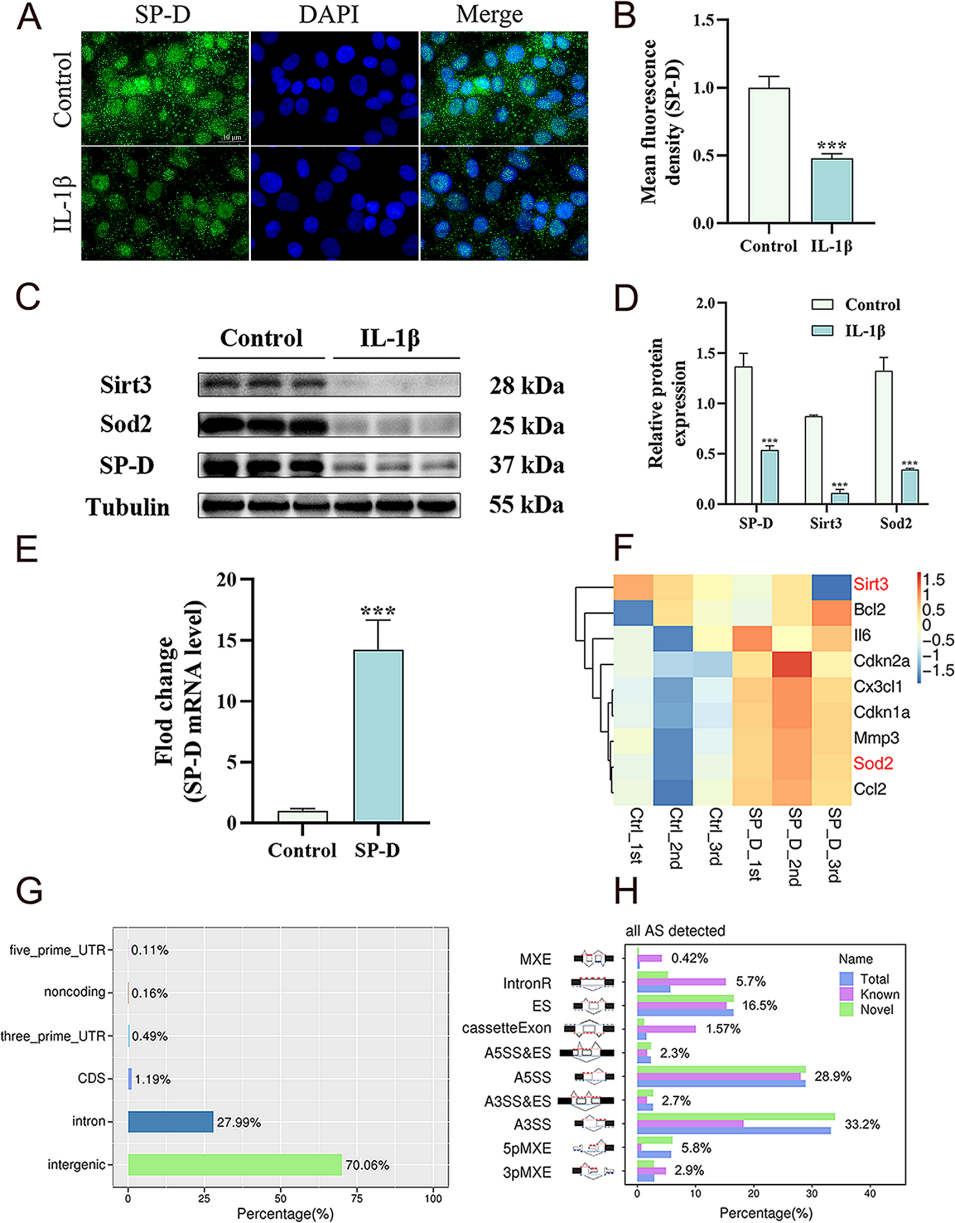
Different expressions of SP-D and SIRT3/SOD2 and SP-D overexpression on the gene expression profile. (**A**, **B**) Senescent chondrocytes were induced by IL-1β for 24 h. Immunofluorescence with antibody to SP-D in chondrocytes. (**C**, **D**) The protein expression of SIRT3, SOD2 and SP-D were assessed via western blotting with beta-tubulin as a loading control. (**E**) Chondrocytes were co-treated with lipofectamine transfected pcDNA3.1 empty plasmid and pcDNA3.1-SP-D plasmid (1 mg/mL). Overexpression efficiency of SP-D was assessed by RT-PCR. (**F**) Heatmap showing high consistency among three replicates of the same groups confirms the correct experimental design and sample sampling. SP-D is significantly associated with the senescence-associated genes. (**G**) Occupancy of different regions in the genomic sequence. (**H**) Percentage of various types of alternative splicing in genomic transcription. Data were expressed as mean ± SEM (*n* = 3). ^***^P < 0.001 vs. the control group

To assess the protein expression differences between normal and senescent chondrocytes, they were treated with IL-1β (10 ng/mL). SP-D expression was detected via immunofluorescence and western blotting. The results revealed a significantly decreased expression of SP-D in senescent chondrocytes than normal chondrocytes (Fig. 1A, B). Furthermore, the expression of SIRT3 and SOD2 proteins, which were associated with antioxidant function was also assessed. The results indicated substantially reduced chondrocyte gene expression of SIRT3 and SOD2 in IL-1β-induced senescent chondrocytes compared to normal chondrocytes (Fig. 1C, D).

#### SP-D overexpression significantly affects the expression of senescence-related chondrocyte’s gene

The pcDNA3.1-SP-D plasmid was transfected into chondrocytes to overexpress SP-D and investigate its controlled targets in OA. Compared with the empty plasmid, pcDNA3.1-SP-D effectively increased SP-D protein production (Fig. 1E). Furthermore, RNA-sequencing analysis was performed to assess the chondrocyte’s gene expression patterns in the SP-D overexpression and the control groups. The activities of DEGs were assessed by GO analysis, and some senescence-associated DEGs were selected for a heatmap plot based on their FPKM values (Fig. 1F). A significant difference was observed between the pcDNA3.1-SP-D overexpression and the control groups and there was high consistency among three replicates of the same groups. Moreover, overexpression of SP-D in chondrocytes was significantly associated with oxidation and senescence-related genes, particularly the SIRT3/SOD2 signaling pathway. The results indicate the occupancy of different genomic regions and the distribution of alternative splicing types during genomic transcription (Fig. 1G, H). These findings represent the diversity of molecular processes and signaling pathways in chondrocytes regulated by SP-D.

### Transfection with rAAV vectors reduced SP-D expression and promoted SASP levels in joint cartilage

**Fig. 2:**
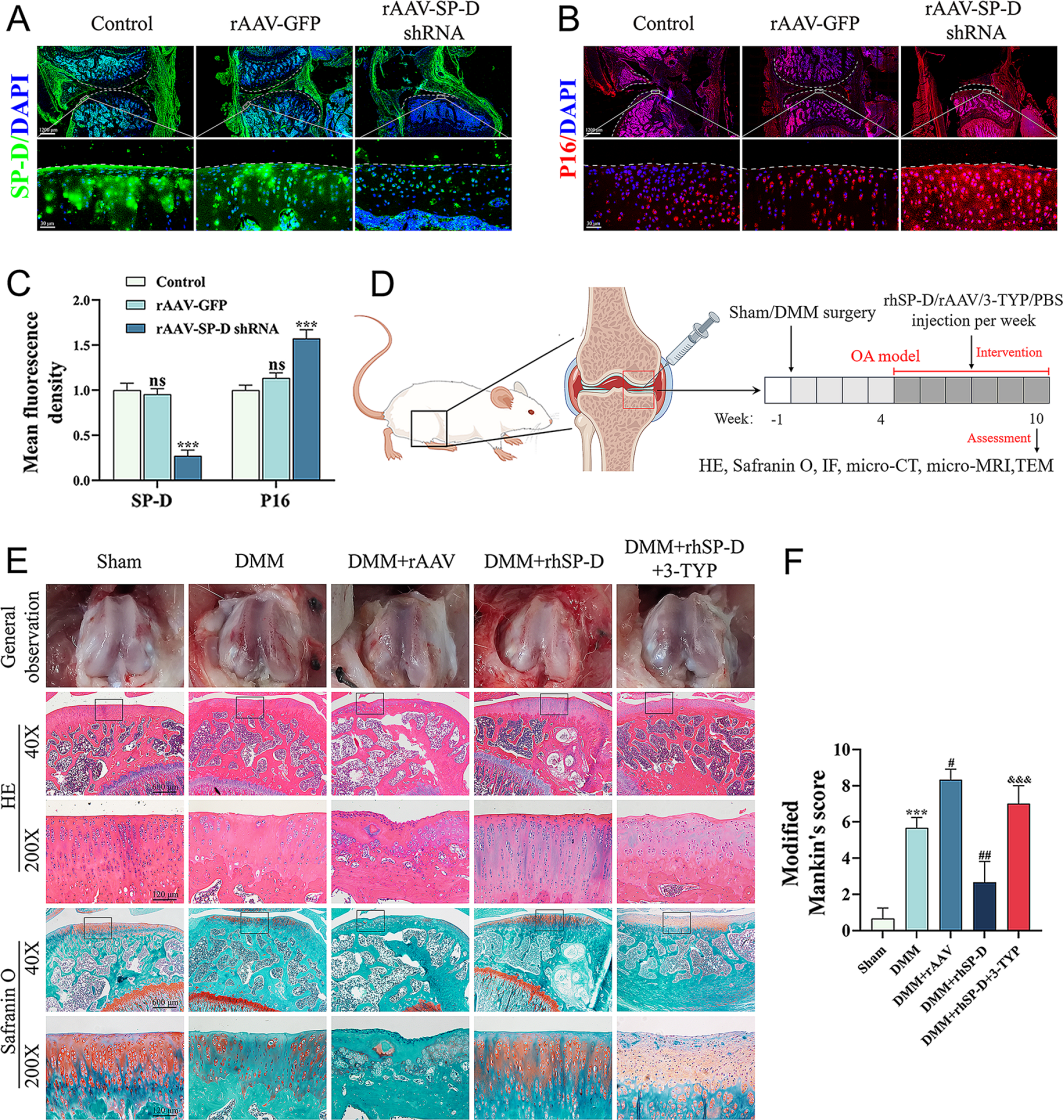
An overview of study timelines and cartilage-protecting effect of SP-D in rat OA model. (**A**) Rats received intra-articularly injections of rAAV encoding SP-D shRNA for 10 consecutive days and were sacrificed at the fourth week after the initial injection. The level of SP-D in cartilage was assessed by immunofluorescence. (**B**) The level of P16 in cartilage tissues was evaluated under the same treatment conditions. (**C**) The mean fluorescence densities of SP-D and P16 were assessed (*n* = 3). (**D**) The rat OA model was established via DMM surgery. At 4 weeks post-surgery, different concentrations of rAAV-SP-D shRNA, rhSP-D and 3-TYP were injected into the right knee joint cavity of rats once peer week. Histological staining, immunofluorescence, Micro-CT, Micro-MRI and TEM were used for detection. (**E**) Macroscopic observation, H&E staining and Safranin-O-Fast Green staining were used to evaluate pathophysiologic morphology of the cartilage at 10 weeks postoperatively. (**F**) The modified Mankin’s scores were assigned to tissue samples. Data were expressed as mean ± SEM (*n* = 5). ^***^P < 0.001 vs. the control and sham operation group; ^#^*P* < 0.05 vs. the OA-induced group; ^&&&^*P* < 0.001 vs. the OA + rhSP-D group

The rAAV-SP-D shRNA was applied to the intra-articular cartilage and its ability to modify SP-D expression in the cartilage was detected. The animals treated with rAAV-SP-D shRNA showed reduced expression of SP-D in the cartilage tissues as compared to the control group (Fig. 2A, C). Furthermore, the expression of SASP in cartilage tissues was also assessed, which revealed that in rAAV-SP-D shRNA-treated cartilage tissues, the P16 level was markedly increased (Fig. 2B, C). These findings imply that by regulating the levels of SASP, SP-D plays a protective role in articular cartilage tissues.

#### In vivo chondroprotective effects of SP-D

Figure 2D displays the timeline for OA modeling, intervention, and sampling, while Fig. 2E indicates the results of macroscopic observations. The surface of the femoral condylar cartilage in the sham group was smooth and without osteochondral formation, while that in the OA-induced group exhibited lesions and a rough eroded surface. Furthermore, the OA-induction group showed enhanced cartilaginous tissue destruction after intra-articular injection of rAAV-SP-D shRNA, and the degree of destruction was improved after rhSP-D injection. However, the ameliorative effect could be counteracted by the SIRT3 inhibitor 3-TYP.

The assessment of the cartilage surface morphology was conducted using H&E and Safranin-O-Fast Green stains. The sham group had a flat cartilage surface which was rich in proteoglycans, while the OA-induced group indicated cartilage defects and hyaline cartilage loss (Fig. 2E). The cartilage degeneration increased more after rAAV-SP-D shRNA injection, as seen by the thickness and surface morphology of the cartilage. In the OA-induced group, treatment with rhSP-D markedly reduced the degree of cartilage degradation, but 3-TYP prevented SP-Dʼs cartilage-protective effects. Furthermore, the OA + rhSP-D group had a lower modified Mankinʼs score, which was higher in the rAAV-SP-D shRNA group as compared to the OA-induced group. However, 3-TYP treatment increased this score in the rhSP-D group (Fig. 2F), suggesting that SP-D could improve cartilage tissue degeneration.

**Fig. 3:**
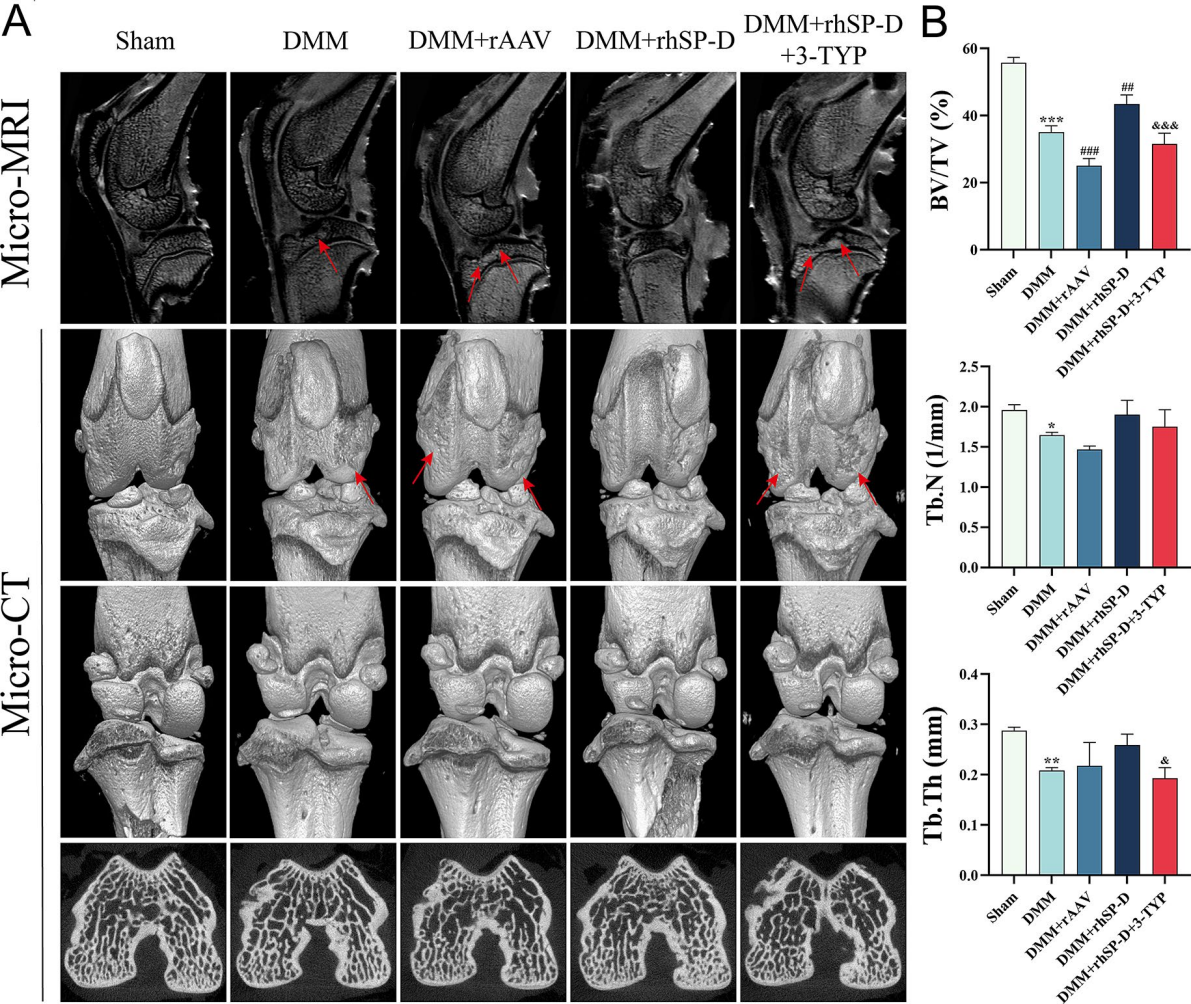
The protective effect of SP-D on cartilage degeneration in rat OA model. (**A**) The knee joints of rats were scanned using Micro-CT and Micro-MRI. (**B**) The bone volume fraction (BV/TV, %), trabecular thickness (Tb. Th, mm) and trabecular number (Tb. N, 1/mm) were analyzed. Data were expressed as mean ± SEM (*n* = 5). ^*^P < 0.05, ^**^P < 0.01 and ^***^P < 0.001 vs. the sham operation group; ^##^*P* < 0.01 and ^###^*P* < 0.001 vs. the OA-induced group; ^&^*P* < 0.05 and ^&&&^*P* < 0.001 vs. the OA + rhSP-D group

The Micro-CT and Micro-MRI scans of the rat’s knee joints were acquired (Fig. 3A). It was revealed that compared with the sham group, the OA-induced group had a significantly decreased bone volume fraction (BV/TV, %), suggesting that there was an osteoporosis and arthroplastic degeneration manifestation of the knee joints in the OA-induced group (Fig. 3B). However, SP-D ameliorated knee joint degeneration in the OA-induced group, suggesting its potential to alleviate cartilage degeneration, and this effect can be antagonized by 3-TYP. Moreover, SP-D silencing with rAAV9 exacerbated cartilage degeneration in the OA-induced group.

#### SP-D alleviates chondrocyte senescence in vivo by upregulating SIRT3/SOD2 pathway

**Fig. 4:**
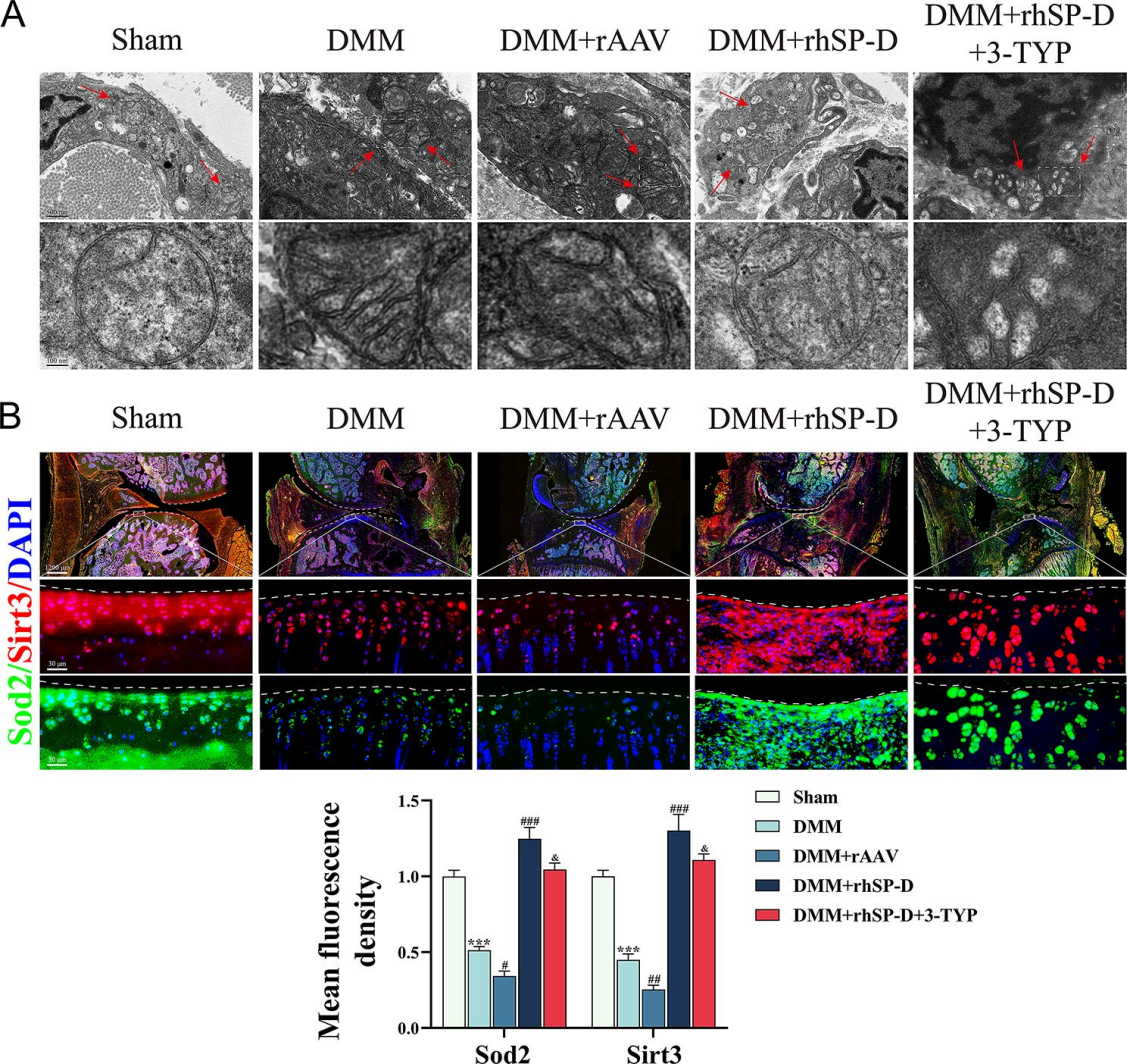
SP-D maintains mitochondrial function in vivo. (**A**) Mitochondrial morphology in synovial tissue was observed and evaluated via TEM. The red arrows represented mitochondria. (**B**) Levels of SOD2 and SIRT3 in each group were assessed by immunofluorescence analysis. Data were expressed as mean ± SEM (*n* = 5). ^***^P < 0.001 vs. the sham operation group; ^#^*P* < 0.05, ^##^*P* < 0.01 and ^###^*P* < 0.001 vs. the OA-induced group; ^&^*P* < 0.05 vs. the OA + rhSP-D group

To investigate the improvement effect of SP-D on mitochondrial function, the mitochondrial morphology in synovial tissues was assessed using TEM (Fig. 4A). The results showed normal mitochondrial morphology of synoviocytes in the sham group, whereas the OA group mitochondria had altered morphology and swelled cristae. Abnormalities in mitochondrial morphology increased even more after rAAV-SP-D shRNA and 3-TYP treatment, while mitochondria in the OA + rhSP-D group improved.

**Fig. 5:**
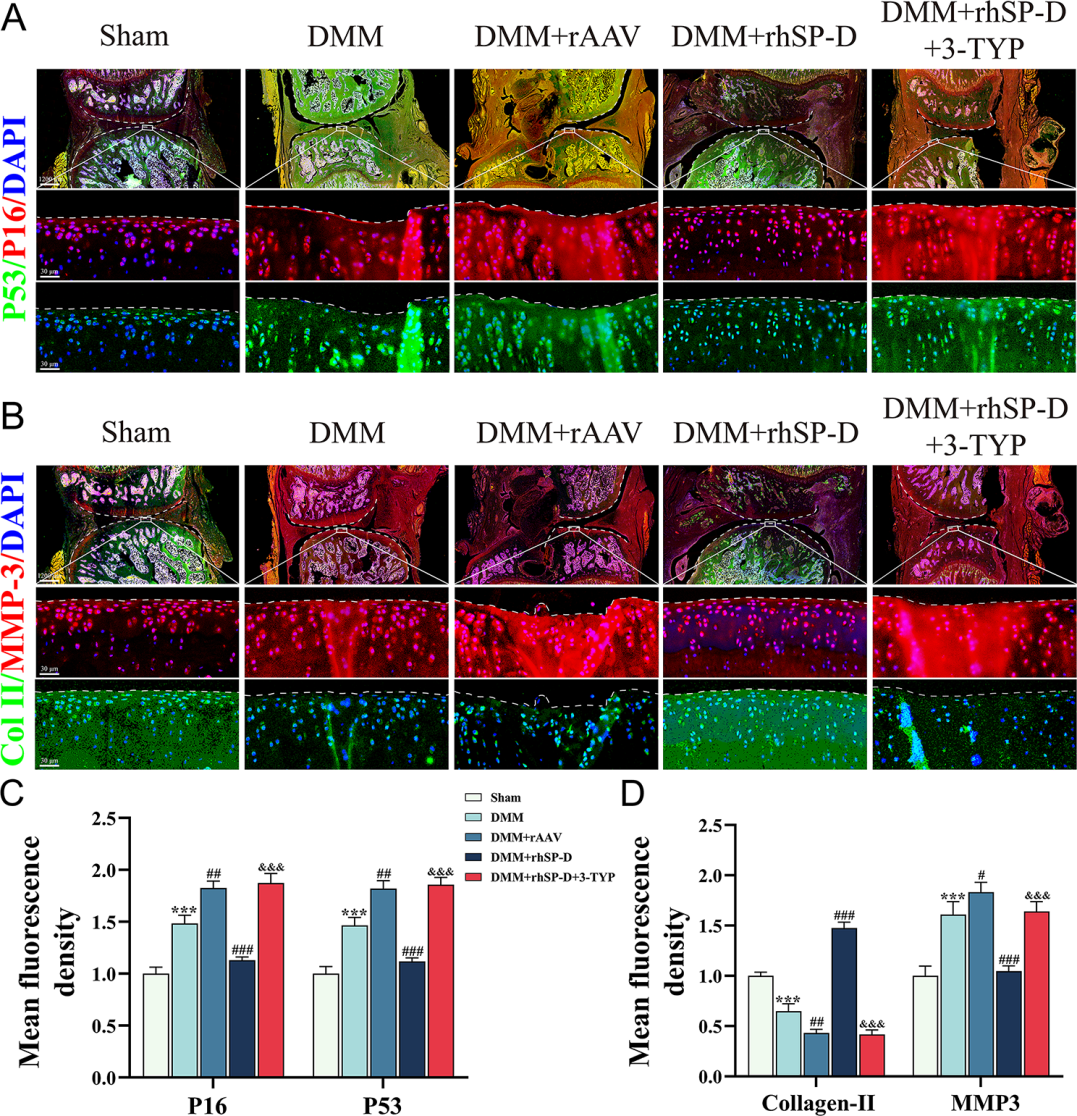
SP-D regulates the expression of SASP in vivo. (**A**) Immunofluorescence with antibodies to P16 and P53 in articular cartilage from the DMM-induced OA rats with rAAV-SP-D shRNA, rhSP-D and 3-TYP treatment at 10 weeks post-surgery. (**B**) Immunofluorescence with antibodies to Collagen-II and MMP3 under the same conditions. Data were expressed as mean ± SEM (*n* = 5). ^***^P < 0.001 vs. the sham operation group; ^#^*P* < 0.05, ^##^*P* < 0.01 and ^###^*P* < 0.001 vs. the OA-induced group; ^&&&^*P* < 0.001 vs. the OA + rhSP-D group

The levels of SOD2, SIRT3, P16, P53, Collagen-II, and MMP3 in the cartilage of each group’s rats were assessed by immunofluorescence (Figs. 4B and 5A and B). It was observed that the expression of P16, P53, and MMP3 was significantly higher while, that of SOD2, SIRT3, and Collagen-II were significantly decreased in the OA-induced group than the sham operation group. Furthermore, rhSP-D decreased the SASP expression, and increased the levels of antioxidant-related proteins in the cartilage of OA rats, suggesting that SP-D could improve the mitochondrial function and reduce the degeneration of cartilage tissue. Moreover, after 3-TYP treatment, the expression levels of SIRT3 and SOD2 slightly decreased, while the expression of SASP significantly increased, suggesting that the therapeutic effect of rhSP-D could be antagonized by 3-TYP. Overall, these results indicated that SP-D could enhance the antioxidant and anti-senescence capacity of cartilage tissues by modulating the SIRT3/SOD2 signaling pathway to suppress SASP expression, thereby alleviating the degeneration of cartilage tissues.

#### SP-D alleviates IL-1β-induced chondrocyte senescence in vitro

**Fig. 6:**
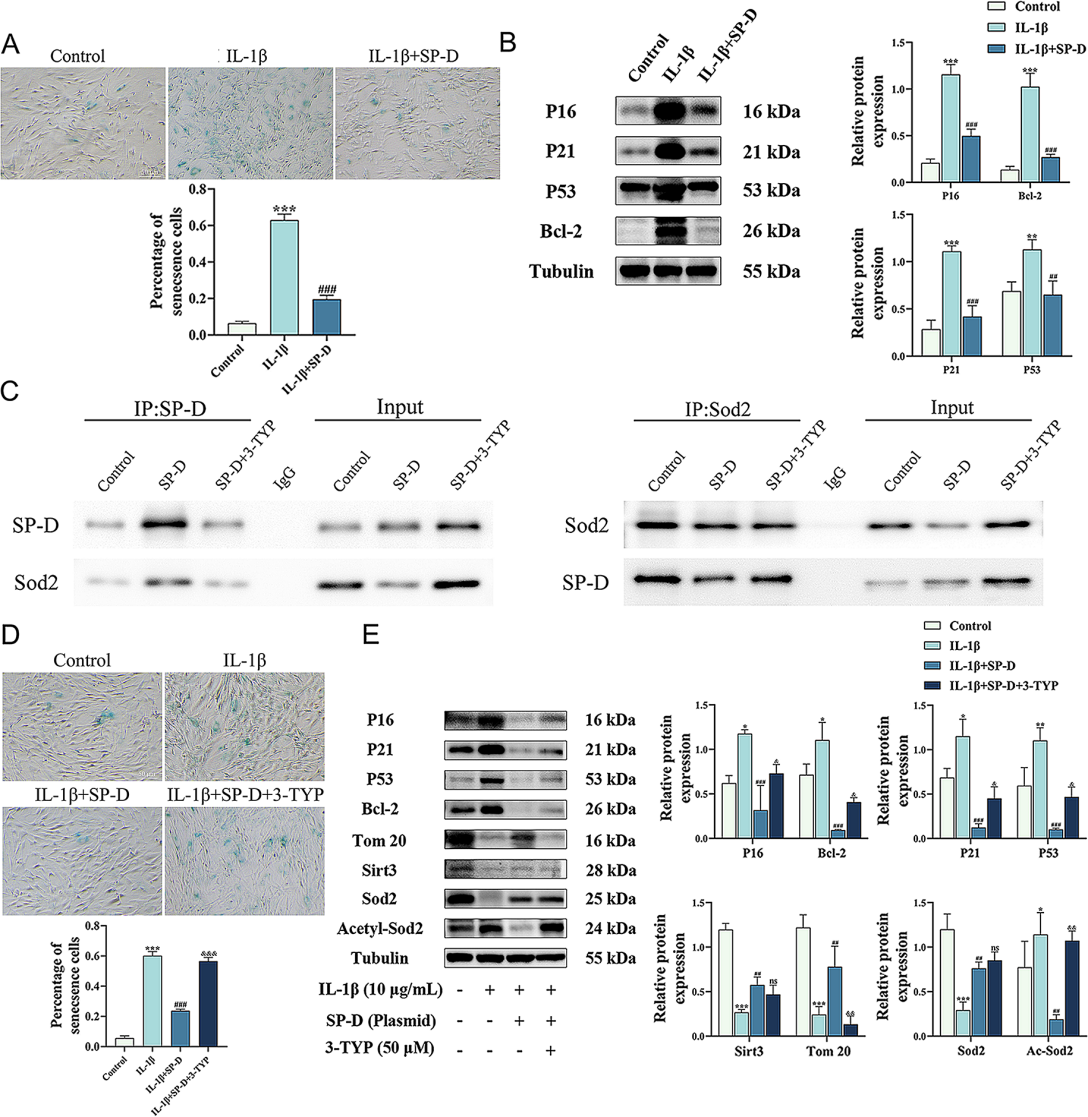
SP-D alleviates chondrocyte senescence by upregulating SIRT3/SOD2 signaling pathway. (**A**) Chondrocytes were transfected using pcDNA3.1-SP-D overexpression plasmid and then incubated with IL-1β for 24 h. The senescent chondrocytes were assessed by SA-β-gal staining. (**B**) The protein expression of P16, P21, P53, and Bcl-2 were assessed via western blotting with beta-tubulin as a loading control. (**C**) Chondrocytes were transfected using pcDNA3.1-SP-D overexpression plasmid, then incubated with 3-TYP for 24 h and total protein was extracted. SP-D and SOD2 were used as bait proteins, respectively, and the interactions between SP-D and SOD2 were verified by Co-IP. (**D**) Chondrocytes were pretreated with 3-TYP for 2 h after SP-D overexpression, followed by co-incubation with IL-1β for 24 h. The senescent chondrocytes in each group were assessed by SA-β-gal staining. (**E**) The protein expression of P16, P21, P53, Bcl-2, Tom20, SIRT3, SOD2, and Ac-SOD2 were assessed via western blotting with beta-tubulin as a loading control. Data were expressed as mean ± SEM (*n* = 3). ^**^P < 0.01 and ***P < 0.001 vs. the control group. ^##^*P* < 0.01 and ^###^*P* < 0.001 vs. the IL-1β group. ns: not significant vs. the IL-1β + SP-D group

To investigate how SP-D alleviates chondrocyte senescence in vitro, the SP-D overexpressing chondrocytes were incubated with IL-1β, and the senescent cells were counted using SA-β-gal staining (Fig. 6A). The results showed a significant reduction in the proportion of SA-β-gal positive cells. Western blotting was performed to evaluate the expression of SASP in senescent chondrocytes, which revealed that compared to the IL-1β group, the levels of P16, P21, P53, and Bcl-2 were significantly reduced in the IL-1β + SP-D group (Fig. 6B). These results indicated that SP-D could alleviate the IL-1β-induced chondrocyte senescence phenotype in vitro.

### SP-D maintains mitochondrial homeostasis by upregulating SIRT3/SOD2 pathway

**Fig. 7:**
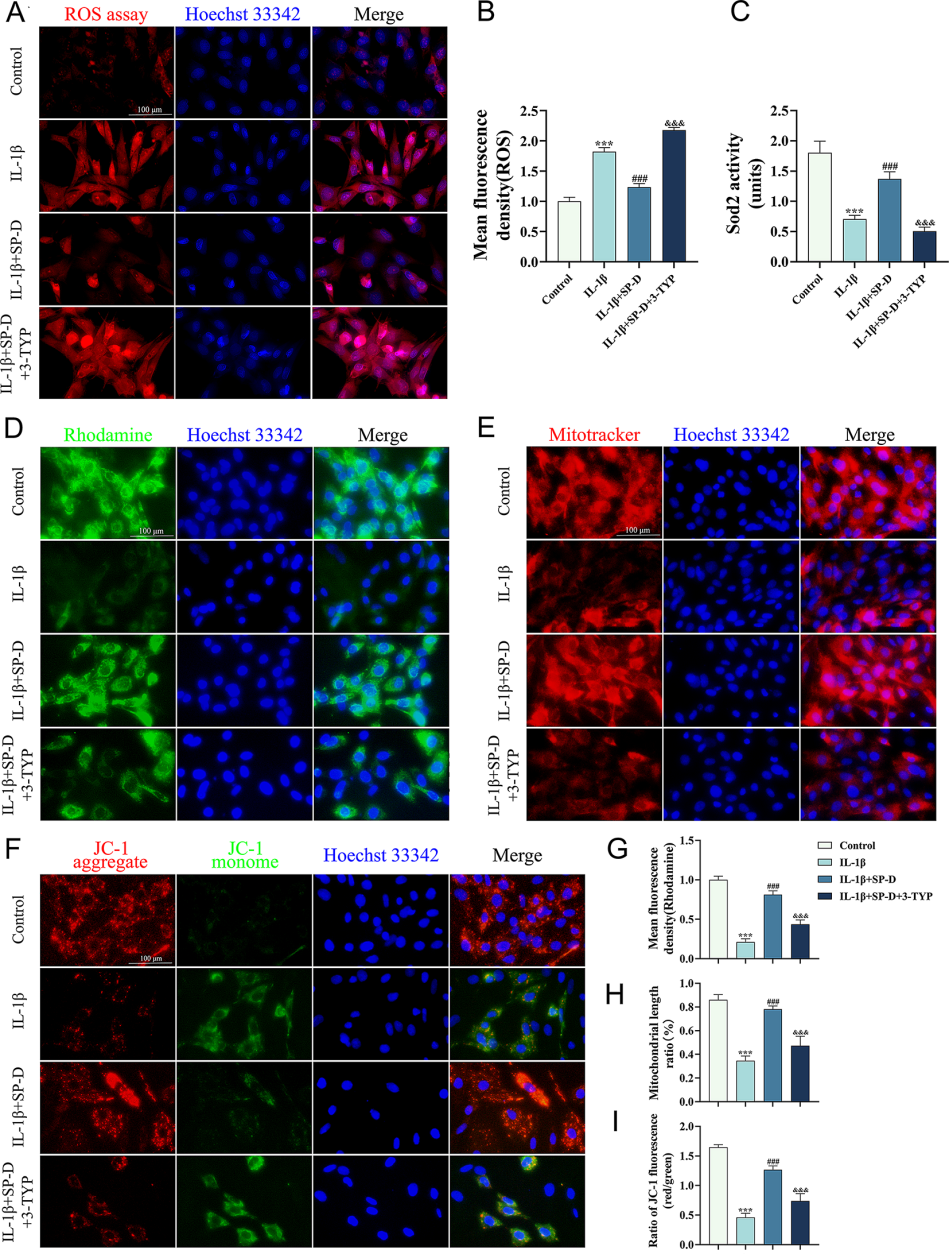
SP-D decreases ROS levels and maintains chondrocyte mitochondrial functional homeostasis. (**A**, **B**) Chondrocytes were pretreated with 3-TYP for 2 h after SP-D overexpression, followed by co-incubation with IL-1β for 24 h. The level of ROS in each group was determined via fluorescence imaging. (**C**) The activity of SOD2 in chondrocytes was determined using a CuZn/Mn-SOD activity assay kit. (**D**) Chondrocytes were pretreated with 3-TYP for 2 h after SP-D overexpression, followed by co-incubation with IL-1β for 24 h. Changes in mitochondrial membrane potential in each group were determined by Rhodamine123 staining. (**E**) Morphology of chondrocyte mitochondria was assessed using MitoTracker Red staining. (**F**) Changes in mitochondrial membrane potential were determined by JC-1 staining. (**G**) Quantitative analysis of chondrocyte mitochondrial membrane potential based on the average fluorescence intensity of Rhodamine123. (**H**) Quantitative analysis of chondrocyte mitochondrial morphology based on MitoTracker Red staining. (**I**) Quantitative analysis of chondrocyte mitochondrial membrane potential based on the ratio of JC-1 fluorescence (red/green). Data were expressed as mean ± SEM (*n* = 3). ***P < 0.001 vs. the control group. ^###^*P* < 0.001 vs. the IL-1β group. ^&&&^*P* < 0.001 vs. the IL-1β + SP-D group

The specific molecular mechanisms by which SP-D alleviates chondrocyte senescence were investigated. As shown in Fig. 6C, SP-D and SOD2 were used as bait proteins, and their interaction was verified through co-immunoprecipitation. Furthermore, immunoblots of another protein complexed with SP-D and SOD2, respectively, were also detected. It was revealed that SP-D and SOD2 could interact with each other intracellularly, consistent with RNA-sequencing results. Then, the SIRT3 inhibitor 3-TYP was utilized for further validation. In the IL-1β-induced chondrocyte senescence model, 3-TYP significantly increased the proportion of SA-β-gal positive cells and the expression of SASP (Fig. 6D, E). The findings suggest that 3-TYP counteracted SP-D’s alleviating effect on cellular senescence. Moreover, 3-TYP significantly decreased SIRT3/SOD2 activity without altering their expression levels and increased the SOD2 acetylation levels (Fig. 6E), thereby elevating intracellular ROS accumulation (Fig. 7A, B). These results suggest that SP-D alleviates chondrocyte senescence by upregulating SIRT3/SOD2 signaling pathway, which can be antagonized by 3-TYP.

Subsequently, the effect of SP-D on mitochondrial function in chondrocytes was explored. Compared with the IL-1β group, SP-D overexpressing chondrocytes had increased expression of the mitochondrial input receptor Tom20 (Fig. 6E) and significantly elevated mitochondrial membrane potential (Fig. 7D, F). In addition, the MitoTracker Red staining indicated that IL-1β-induced abnormal mitochondrial morphology could be counteracted by SP-D (Fig. 7E). The mitochondrial morphology observed in senescent chondrocytes was ameliorated after SP-D overexpression treatment. Furthermore, after 3-TYP-mediated inhibition of SIRT3, mitochondrial length was reduced again. Overall, these data suggested that SP-D can preserve mitochondrial function by upregulating SIRT3/SOD2 signaling pathway, thereby alleviating chondrocyte senescence.

## Discussion

OA is a significant contributor to disability in the elderly and its prevalence is increasing annually with the aging population (Peat and Thomas 2021; Sherwood 2019). Currently, there are limited therapeutic options for OA and clinical treatment is primarily focused on conservative treatments to alleviate local symptoms and inhibit disease progression. In advanced stages, patients often resort to joint replacement surgery to restore joint function (Cho et al. 2021). Therefore, the identification of novel therapeutic targets that could effectively inhibit OA progression and ameliorate the local inflammatory milieu is essential. This study investigated the effects of SP-D on chondrocyte senescence and mitochondrial homeostasis by modulating SIRT3/SOD2 signaling pathway. Furthermore, the role of SP-D in mitigating articular cartilage degeneration was elucidated using the rat OA model.

The primary pathological features of OA encompass degenerative alterations and aberrant proliferation of chondrocytes. Senescent chondrocytes secrete and accumulate SASP components extensively, which induces senescence in the neighboring healthy chondrocytes via paracrine effects. Therefore, cellular senescence plays an essential role in OA initiation and progression (Liu et al. 2023; Riegger et al. 2023; Toh et al. 2016). SP-D, a soluble C-type lectin, is significantly expressed in intra- and extra-pulmonary tissues, playing a crucial role in organisms’ defense and innate immunity (Madan & Kishore 2020, Mahajan et al. 2014). Our previous research has revealed significantly diminished expression of SP-D in chondrocytes derived from OA patients compared to normal chondrocytes (Jiang et al. 2022; Zhou et al. 2020). To elucidate how SP-D is associated with OA, RNA-sequencing of SP-D overexpressing chondrocytes and controls, followed by bioinformatics analysis was conducted to identify enriched differential genes and signaling pathways. It was observed that SP-D overexpression significantly affects oxidation- and senescence-associated genes, particularly impacting SIRT3/SOD2 signaling pathway. This discovery offers valuable insights for further exploration of the specific molecular mechanisms underlying SP-D regulation of OA.

The in vitro experiments validated that in the IL-1β induced chondrocyte senescence model, the expression levels of SP-D, SIRT3, and SOD2 were markedly reduced. Furthermore, SOD2 functions as a principal antioxidant enzyme in the mitochondria and is involved in scavenging intracellularly accumulated ROS and mitigating cellular senescence (Miao and St Clair 2009). Based on these findings, it was hypothesized that SP-D may alleviate OA progression by mitigating chondrocyte senescence via intracellular SIRT3/SOD2 signaling pathway upregulation.

To investigate the regulatory role of SP-D in cellular senescence in vivo, SP-D-silenced rAAV9 was generated. Injection of rAAV9 into the rat knee joint cavity significantly diminished SP-D expression in articular cartilage and promoted SASP secretion. Furthermore, rat OA models were established using DMM surgery to further probe the regulatory function of SP-D in OA. The DMM surgery-induced OA model inflicts a milder degree of injury, which resembles early clinical OA condition, and better mimics its pathological characteristics of early OA human knee, rendering it more clinically relevant (Glasson et al. 2007). Furthermore, SP-D levels in cartilage tissue were modulated by joint cavity injection of rAAV9 or rhSP-D. Reduced SP-D levels caused aberrant changes in mitochondrial morphology in synovial tissue, downregulated antioxidant-related genes (SIRT3 and SOD2), and upregulated SASP secretion in cartilage tissue. Elevated SASP levels in tissues exacerbated articular cartilage degeneration, resulting in further OA development, as evidenced by microscopic and imaging manifestations. These data indicated that endogenous SP-D attenuates cellular senescence and inhibits articular cartilage degeneration by modulating mitochondrial homeostasis and redox balance in tissues. This, in turn, diminishes OA onset and progression, underscoring its potential therapeutic value in OA management.

The role of SP-D in alleviating cellular senescence in the IL-1β-induced chondrocyte senescence model was explored. In senescent cells, the accumulation of cell cycle-associated factors such as P16 and P21 perpetuates, leading to sustained activation of retinoblastoma (RB) family proteins that inhibit the trans-activation of E2F and result in irreversible cell cycle arrest (Huang et al. 2022; Salama et al. 2014). Furthermore, senescent cells continuously secrete SASP, which mediates non-cell-autonomous senescence effects, thereby contributing to senescence-associated tissue damage and degeneration, ultimately fostering OA progression (Coryell et al. 2021; Jeon et al. 2018; Liu et al. 2022; McCulloch et al. 2017). Therefore, molecules capable of mitigating IL-1β-induced cellular senescence can serve as therapeutic candidates for OA. It was observed that SP-D treatment reduced the proportion of SA-β-gal positive cells and diminished the protein expression of senescence-related factors P16, P21, P53, and Bcl-2 in chondrocytes. Moreover, in chondrocytes, SP-D improved mitochondrial morphology, preserved mitochondrial membrane potential, and enhanced SOD2 activity, thereby reducing ROS accumulation. However, these effects were antagonized by 3-TYP, a SIRT3 inhibitor. Altogether, these findings suggest that SP-D enhances intracellular mitochondrial function and inhibits chondrocyte senescence possibly by upregulating SIRT3/SOD2 signaling pathway.

**Fig. 8:**
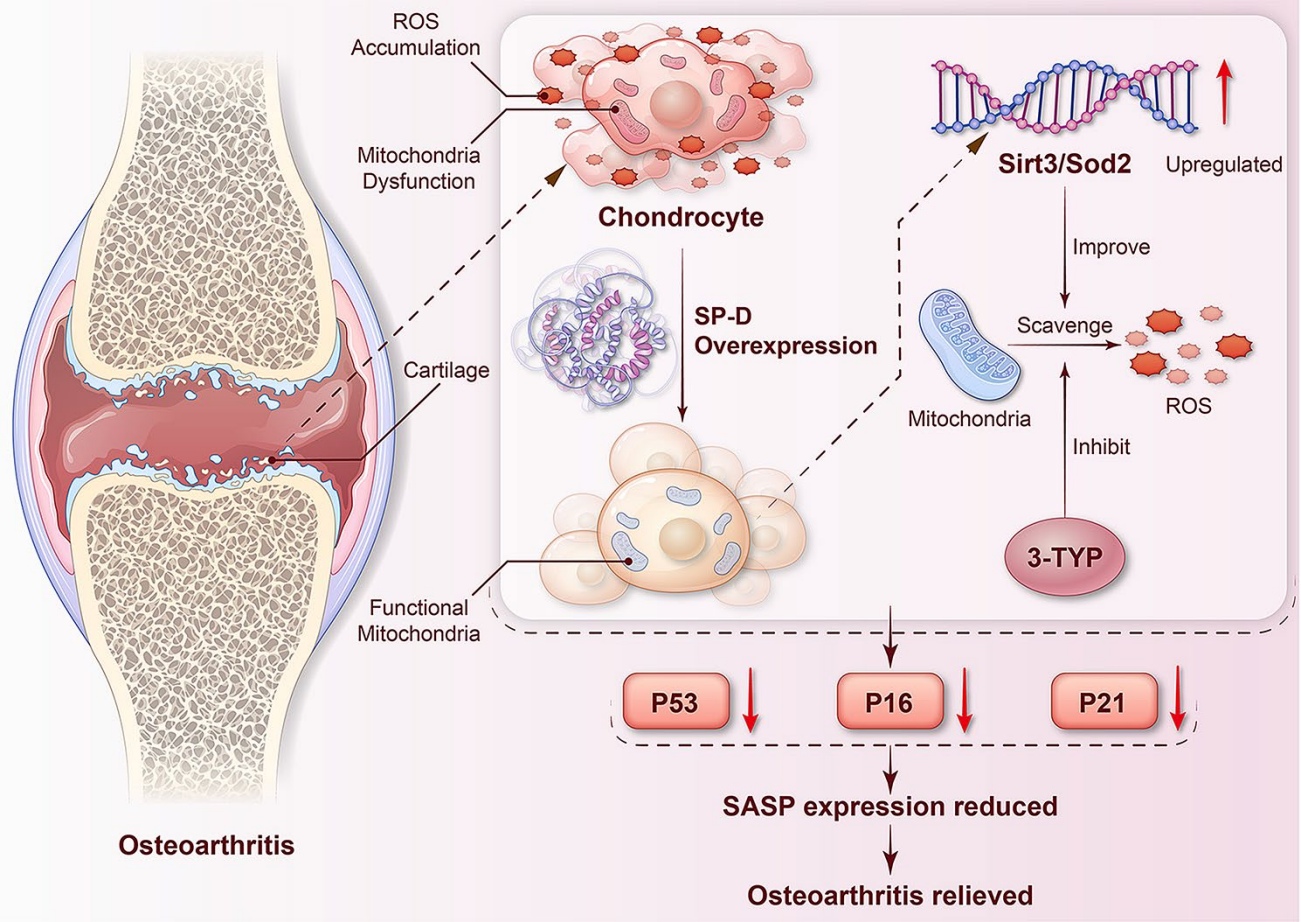
Cartoon diagram showing the molecular mechanism hypothesis that SP-D alleviates chondrocyte senescence by upregulating SIRT3/SOD2 signaling pathway

Oxidative stress is an essential factor in chondrocyte senescence, with intracellular mitochondrial dysfunction and diminished SIRT3/SOD2 activity being significant contributors to intracellular redox imbalance and ROS accumulation. It has been indicated that increased intracellular ROS levels can activate the NF-κB pathway, which enhances the synthesis of inflammatory cytokines and matrix-degrading enzymes, thereby promoting synovial cartilage inflammation, cartilage matrix degradation, and OA progression (Freund et al. 2010; Goldring and Marcu 2009; Shakibaei et al. 2007). Here, SP-D ameliorated articular cartilage degeneration enhanced the state of chondrocyte senescence and preserved mitochondrial function homeostasis. However, these effects were counteracted by 3-TYP. Based on these data it was hypothesized that SP-D may bind to SOD2 and promote mitochondrial function by upregulating SIRT3/SOD2 signaling pathway, thereby reducing excessive intracellular ROS and oxidative stress events, ultimately alleviating chondrocyte senescence and conferring protective effects on articular cartilage, thus impeding OA progression (Fig. 8). Although it has been verified that SP-D exerts its antioxidant and anti-aging effects by modulating SIRT3/SOD2 signaling pathway, further studies are required to assess its potential upstream and downstream cascade effects. Moreover, future studies should incorporate SP-D knockout mice to further delineate the critical role of SP-D in OA pathogenesis. Furthermore, while the DMM model is widely used to study OA pathogenesis, it primarily reflects post-traumatic OA rather than age-related OA. Given the heterogeneity of OA, with contributions from aging, trauma, and inflammation, future studies should incorporate naturally aging models to provide a more comprehensive understanding of SP-D’s role across different OA subtypes.

This study also observed SP-D expression on the surface of cartilage tissue. Upon exogenous suppression of SP-D, the expression of SASP factors on the cartilage surface increased, but no significant alteration in cartilage tissue morphology was observed. It was postulated that diminished SP-D expression renders the cartilage tissue in a sub-healthy state, wherein the secretion level within the cartilage is affected, thereby altering the cellular microenvironment. However, this change in the cartilage morphology might not be observed immediately. Furthermore, cartilage tissue stimulation by other factors, such as DMM surgery, may exacerbate cartilage degeneration and augment SASP factor expression. Hence, SP-D could potentially function as a protective agent in cartilage tissue. With age, alterations in the senescent cell’s secretion level gradually reduce endogenous SP-D, which diminishes its protective effect. Coupled with the impact of external abnormal mechanical stress and the establishment of a local inflammatory microenvironment, these factors collectively contribute to the onset and progression of OA. Therefore, the maintenance of SP-D levels and its protective function in cartilage tissue could potentially decelerate OA progression, offering a novel target for OA prevention and treatment.

## Conclusion

In conclusion, the study revealed the effect of SP-D-mediated down-regulation of senescence in IL-1β-induced chondrocytes and DMM-induced cartilage degeneration. Furthermore, it also identified that SP-D alleviates age-associated pathological changes via the SIRT3/SOD2 pathway. These data indicate that targeting SP-D and the SIRT3/SOD2 pathway might be a promising therapeutic strategy for OA.

## Electronic supplementary material

Below is the link to the electronic supplementary material.

Supplementary Material 1

## Data Availability

No datasets were generated or analysed during the current study.

## Abbreviations

OA: Osteoarthritis
SA-β-gal: Senescence-associated β-galactosidase
SASP: Senescence-associated secretory phenotype
ECM: Extracellular matrix
SOD2: Superoxide dismutase 2
ROS: Reactive oxygen species
SIRT3: Sirtuin 3
NF-κB: Nuclear factor kappa-B
DMM: Destabilization of the medial meniscus
rhSP-D: Recombinant human SP-D
IL-1β: Interleukin-1 beta
RT-PCR: Reverse transcription-polymerase chain reaction
FPKM: Fragments per kilobase of transcript per million fragments mapped
DEGs: Differential expression genes
SD: Sprague-Dawley
H&E: Hematoxylin-Eosin
TEM: Transmission electron microscope
Co-IP: Coimmunoprecipitation
ANOVA: One-way analysis of variance
