# Supplementary figures and images for: Surfactant protein D alleviates chondrocytes senescence by upregulating SIRT3/SOD2 pathway in osteoarthritis
Source: Mol Med. 2025 Apr 30;31:161. doi: 10.1186/s10020-025-01221-6 (PMC12044875; doi:10.1186/s10020-025-01221-6)

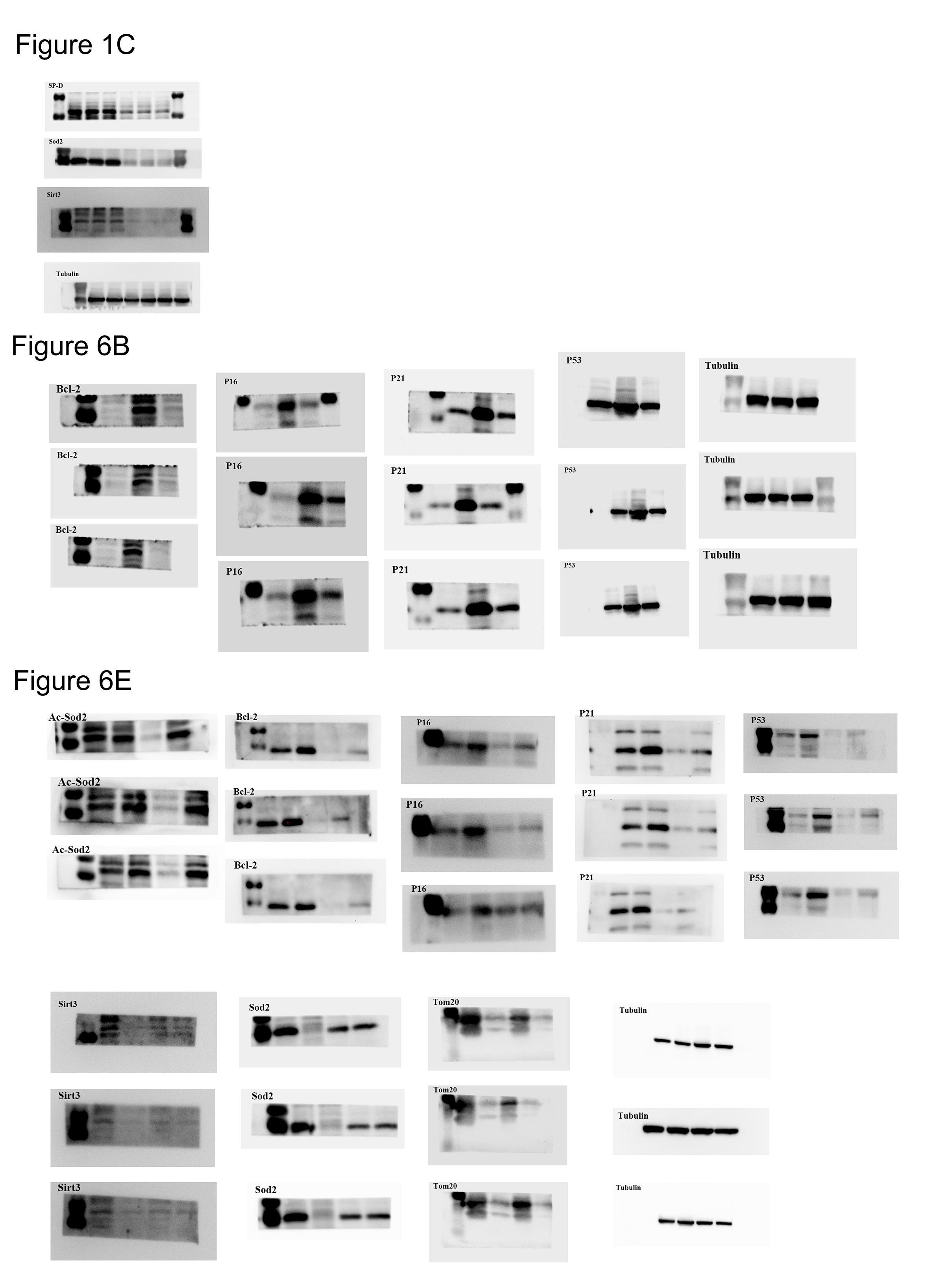

Supplement: Supplementary file 1 — Supplementary Material 1 [file 10020_2025_1221_MOESM1_ESM.tif]
